# Supplementary material for: Exploration driven by a medial preoptic circuit facilitates fear extinction in mice
Source: Commun Biol. 2023 Jan 27;6:106. doi: 10.1038/s42003-023-04442-9 (PMC9883483; doi:10.1038/s42003-023-04442-9)
Supplement: Supplementary file 2 — Supplementary Information [file 42003_2023_4442_MOESM2_ESM.pdf]

Supplementary information

Exploration driven by a medial preoptic circuit facilitates fear extinction in mice

Anna Shin[1, 2†], Jia Ryoo[1, 2†], Kwanhoo Shin[1, 2], Junesu Lee[1, 2], Seohui Bae[3], Dae-Gun Kim[1, 2, 4], Sae-Geun Park[1], Daesoo Kim[1, 2, a*]

[1] Department of Biological Sciences, KAIST, Daejeon 34141, Republic of Korea

[2] Department of Brain and Cognitive Sciences, KAIST, Daejeon 34141, Republic of Korea

[3] KAIST Institute of Artificial Intelligence, KAIST, Daejeon 34141, Republic of Korea

[4] ACTNOVA, Daejeon 34109, Republic of Korea

[†] These authors contributed equally: Anna Shin, Jia Ryoo.

[a] Lead Contact

*Correspondence: daesoo@kaist.ac.kr (D. Kim).

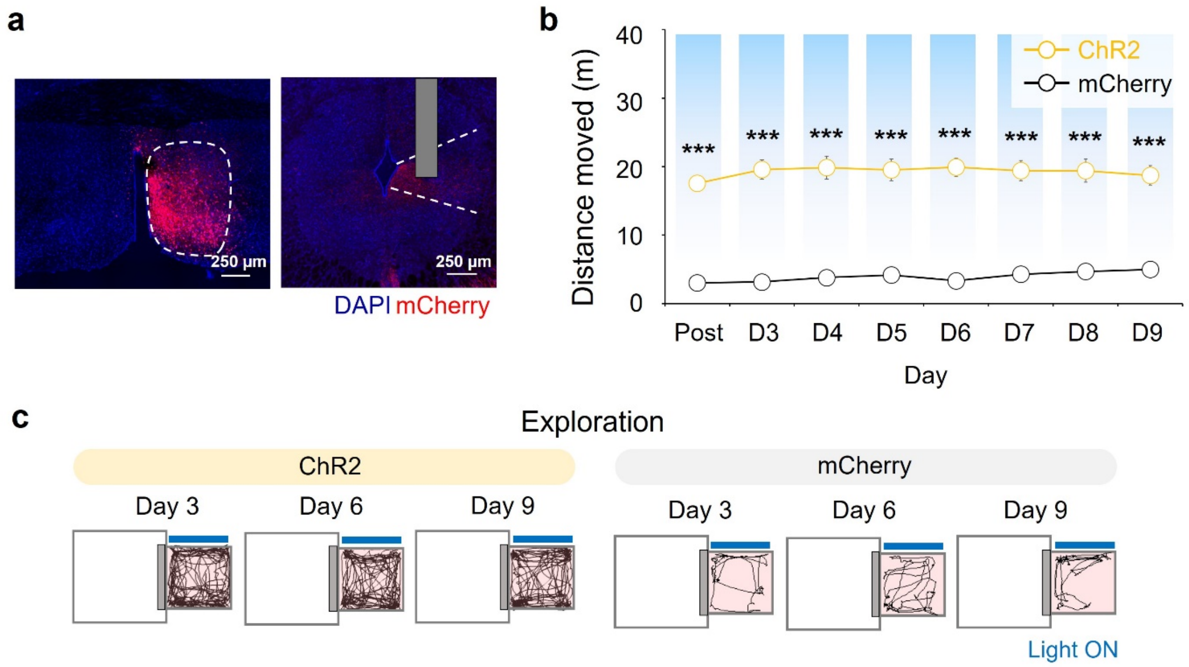
a
250 µm
250 µm
DAPI mCherry
b
Distance moved (m)
40
30
20
10
0
ChR2
mCherry
***
Post
D3
D4
D5
D6
D7
D8
D9
Day
c
Exploration
ChR2
mCherry
Day 3
Day 6
Day 9
Light ON

Supplementary Figure. 1: Photostimulation of the MPA-vPAG circuit increases exploration in the fear-conditioned zone. (a) A representative image of AAV2.5-CaMKIIα-mCherry expression (red) in the MPA and the vPAG. Scale bar: 250 μm. (b) Distance moved in the shock zone during the exploration test. The ChR2 group (yellow, n = 8) and the mCherry group (black, n = 8; two-way RM ANOVA test, Holm-Sidak post-hoc analysis). (c) A representative tracking the center points of mice during exploration. ***$p < 0.001$. All error bars represent s.e.m. Further information on statistical analyses is given in the supplementary data 1.

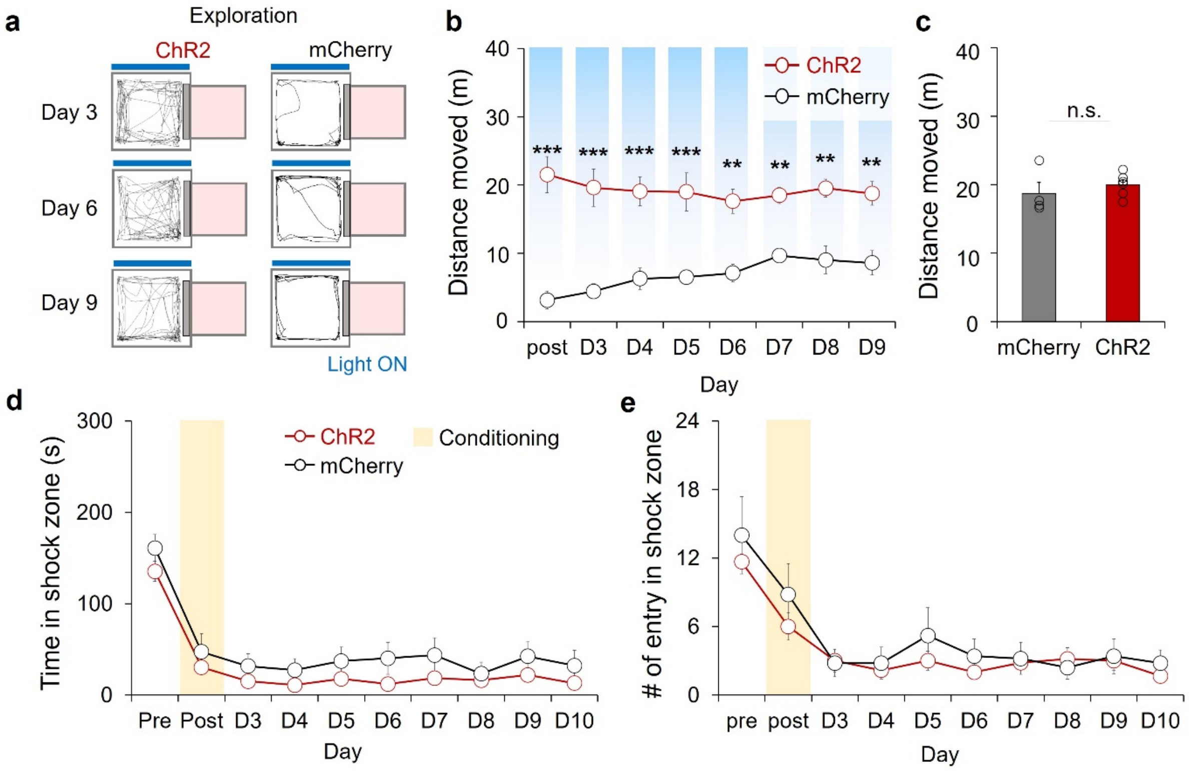
a
Exploration
ChR2
mCherry
Day 3
Day 6
Day 9
Light ON
b
Distance moved (m)
ChR2
mCherry
***
***
***
***
**
**
**
**
post
D3
D4
D5
D6
D7
D8
D9
Day
c
Distance moved (m)
n.s.
mCherry
ChR2
d
Time in shock zone (s)
ChR2
mCherry
Conditioning
Pre
Post
D3
D4
D5
D6
D7
D8
D9
D10
Day
e
# of entry in shock zone
pre
post
D3
D4
D5
D6
D7
D8
D9
D10
Day

Supplementary Figure. 2: Increased exploration induced by photostimulation of the MPA-vPAG circuit in the safe zone does not promote fear extinction. (a) A representative tracking the center points of mice during exploration. (b) Distance moved in the safe zone during the exploration test. The ChR2 group (red, n = 6) and the mCherry group (black, n = 4; two-way RM ANOVA test, Holm-Sidak post-hoc analysis). (c) Distance moved in both zones during extinction test for the mCherry (n = 4) and the ChR2 group (n = 6; unpaired *t*-test). (d) Time in a safe zone during the exploration test. The ChR2 group (red, n = 6) and mCherry group (black, n =4; two-way RM ANOVA test, Holm-Sidak post-hoc analysis). (e) The number of entries into the shock zone during the extinction test. The ChR2 group (red, n = 6) and mCherry group (black, n =4; two-way RM ANOVA test, Holm-Sidak post-hoc analysis). n.s., not significant as $p > 0.05$. $^{**}p < 0.01$, $^{***}p < 0.001$. All error bars represent s.e.m. Further information on statistical analyses is given in the supplementary data 1.

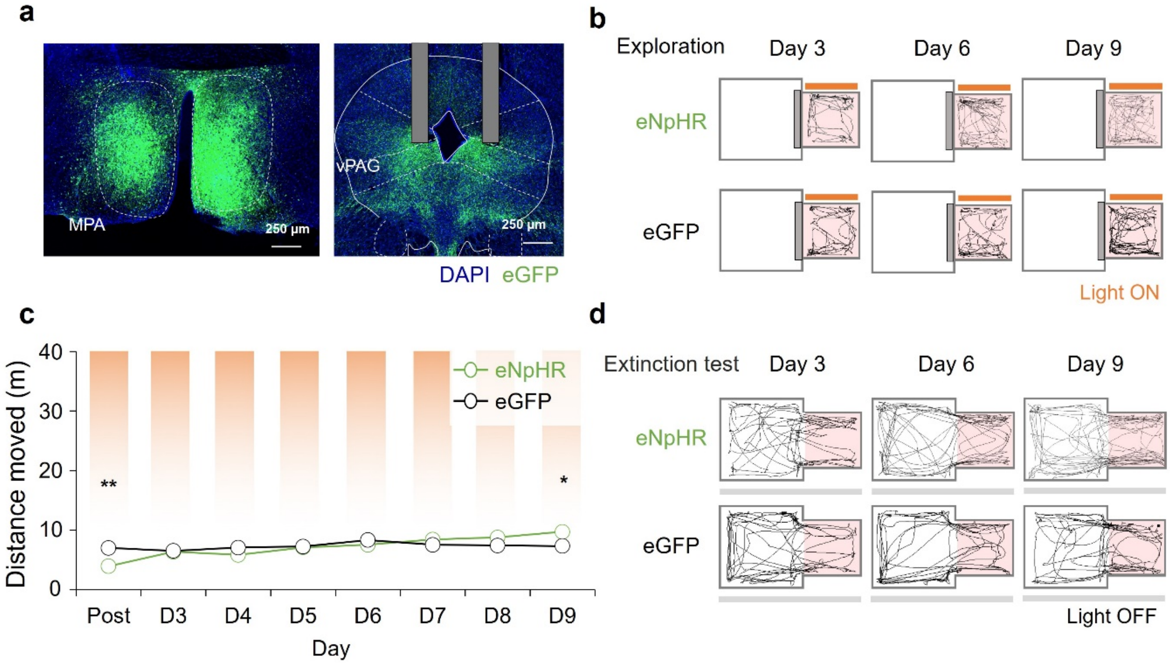

a
MPA
250 µm
vPAG
250 µm
DAPI eGFP
b
Exploration
Day 3
Day 6
Day 9
eNpHR
eGFP
Light ON
c
Distance moved (m)
40
30
20
10
0
**
*
eNpHR
eGFP
Post
D3
D4
D5
D6
D7
D8
D9
Day
d
Extinction test
Day 3
Day 6
Day 9
eNpHR
eGFP
Light OFF

Supplementary Figure. 3: Photoinhibition of the MPA-vPAG circuit does not affect exploration. (a) A representative image of AAV2/5-CaMKIIα-eGFP expression (green) in the MPA and in the vPAG. Scale bar: 250 μm. (b) A representative tracking the center points of mice during exploration in the shock zone. (c) Distance moved in the safe zone during the exploration test. The eNpHR group (green, n = 7) and the eGFP group (black, n = 6; two-way RM ANOVA test, Holm-Sidak post-hoc analysis). (d) A representative tracking the center points of mice during extinction in both zones. *$p < 0.05$, **$p < 0.01$. All error bars represent s.e.m. Further information on statistical analyses is given in the supplementary data 1.

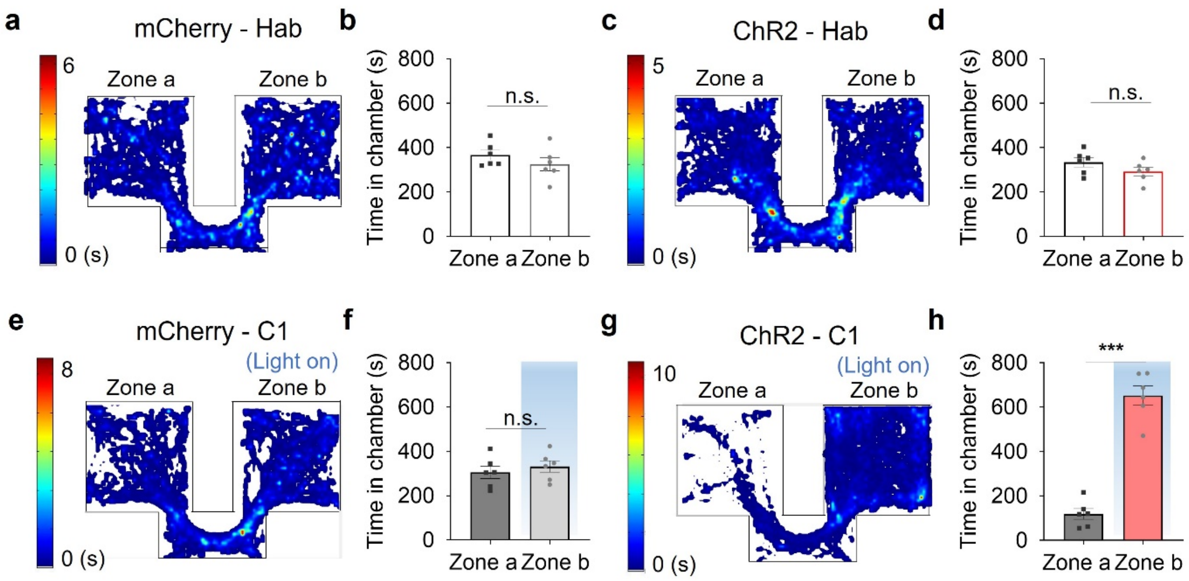
a
mCherry - Hab
6
0 (s)
Zone a
Zone b
b
Time in chamber (s)
800
600
400
200
0
n.s.
Zone a
Zone b
c
ChR2 - Hab
5
0 (s)
Zone a
Zone b
d
Time in chamber (s)
800
600
400
200
0
n.s.
Zone a
Zone b
e
mCherry - C1
(Light on)
8
0 (s)
Zone a
Zone b
f
Time in chamber (s)
800
600
400
200
0
n.s.
Zone a
Zone b
g
ChR2 - C1
(Light on)
10
0 (s)
Zone a
Zone b
h
Time in chamber (s)
800
600
400
200
0
***
Zone a
Zone b

Supplementary Figure. 4: Place-preference chamber exploration before and during MPA-vPAG circuit activation. (a) A representative heat map of mouse center point locations during the habituation session. (b) Time in zone a and zone b for the mCherry group during the habituation session (n = 6; paired two-tailed *t*-test). (c) A representative heat map of mouse center point locations during the habituation session. (d) Time in zone a and zone b for the ChR2 group during the habituation session (n = 6; paired two-tailed *t*-test). (e) A representative heat map of mouse center point locations during day-1 of conditioning. (f) Time in zone a and zone b for the mCherry group during day-1 conditioning (n = 6; paired two-tailed *t*-test). (g) A representative heat map of mouse center point locations during day-1 of conditioning. (h) Time in zone a and zone b for the ChR2 group during day-1 conditioning (n = 6; paired two-tailed *t*-test). ***$p < 0.001$; n.s., not significant as $p > 0.05$. All error bars represent s.e.m. Further information on statistical analyses is given in the supplementary data 1.

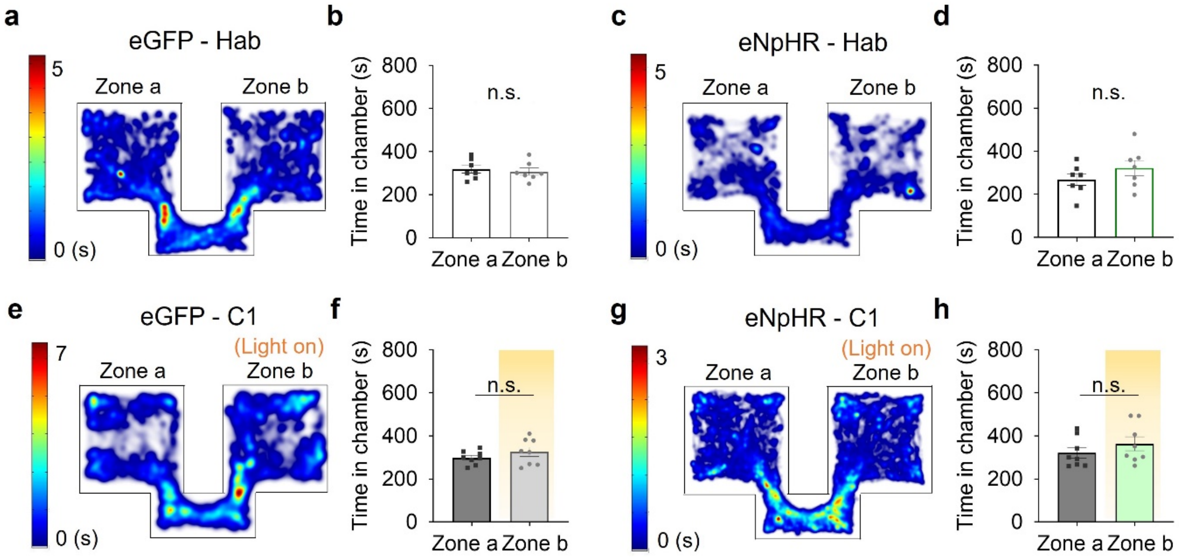

a
eGFP - Hab
5
0 (s)
Zone a
Zone b
b
Time in chamber (s)
800
600
400
200
0
n.s.
Zone a
Zone b
c
eNpHR - Hab
5
0 (s)
Zone a
Zone b
d
Time in chamber (s)
800
600
400
200
0
n.s.
Zone a
Zone b
e
eGFP - C1
7
0 (s)
(Light on)
Zone a
Zone b
f
Time in chamber (s)
800
600
400
200
0
n.s.
Zone a
Zone b
g
eNpHR - C1
3
0 (s)
(Light on)
Zone a
Zone b
h
Time in chamber (s)
800
600
400
200
0
n.s.
Zone a
Zone b

Supplementary Figure. 5: Place-preference chamber exploration before and during MPA-vPAG circuit inactivation. (a) A representative heat map of mouse center point locations during the habituation session. (b) Time in zone a and zone b for the eGFP group during the habituation session (n = 7; paired two-tailed *t*-test). (c) A representative heat map of mouse center point locations during the habituation session. (d) Time in zone a and zone b for the eNpHR group during the habituation session (n = 8; paired two-tailed *t*-test). (e) A representative heat map of mouse center point locations during day-1 of conditioning. (f) Time in zone a and zone b for the eGFP group during day-1 of conditioning (n = 7; paired two-tailed *t*-test). (g) A representative heat map of mouse center point locations during day-1 of conditioning. (h) Time in zone a and zone b for the eNpHR group during day-1 of conditioning (n = 8; paired two-tailed *t*-test). n.s., not significant as $p > 0.05$. All error bars represent s.e.m. Further information on statistical analyses is given in the supplementary data 1.
