# Supplementary material for: Exploration driven by a medial preoptic circuit facilitates fear extinction in mice
Source: Commun Biol. 2023 Jan 27;6:106. doi: 10.1038/s42003-023-04442-9 (PMC9883483; doi:10.1038/s42003-023-04442-9)
Supplement: Supplementary file 3 — Description of Additional Supplementary Files [file 42003_2023_4442_MOESM3_ESM.pdf]

Description of Additional Supplementary Files

File Name: Supplementary Data 1
Description: Statistical comparisons and source data underlying figures.
