# Supplementary material for: Exploration driven by a medial preoptic circuit facilitates fear extinction in mice
Source: Commun Biol. 2023 Jan 27;6:106. doi: 10.1038/s42003-023-04442-9 (PMC9883483; doi:10.1038/s42003-023-04442-9)
Supplement: Supplementary file 5 — Reporting Summary [file 42003_2023_4442_MOESM5_ESM.pdf]

Corresponding author(s): Daesoo Kim

Last updated by author(s): Jan 9, 2023

Reporting Summary

Nature Portfolio wishes to improve the reproducibility of the work that we publish. This form provides structure for consistency and transparency in reporting. For further information on Nature Portfolio policies, see our Editorial Policies and the Editorial Policy Checklist.

Statistics

For all statistical analyses, confirm that the following items are present in the figure legend, table legend, main text, or Methods section.

n/a	Confirmed	
☐	☒	The exact sample size (*n*) for each experimental group/condition, given as a discrete number and unit of measurement
☐	☒	A statement on whether measurements were taken from distinct samples or whether the same sample was measured repeatedly
☐	☒	The statistical test(s) used AND whether they are one- or two-sided<br>*Only common tests should be described solely by name; describe more complex techniques in the Methods section.*
☐	☒	A description of all covariates tested
☐	☒	A description of any assumptions or corrections, such as tests of normality and adjustment for multiple comparisons
☐	☒	A full description of the statistical parameters including central tendency (e.g. means) or other basic estimates (e.g. regression coefficient) AND variation (e.g. standard deviation) or associated estimates of uncertainty (e.g. confidence intervals)
☐	☒	For null hypothesis testing, the test statistic (e.g. *F*, *t*, *r*) with confidence intervals, effect sizes, degrees of freedom and *P* value noted<br>*Give P values as exact values whenever suitable.*
☒	☐	For Bayesian analysis, information on the choice of priors and Markov chain Monte Carlo settings
☒	☐	For hierarchical and complex designs, identification of the appropriate level for tests and full reporting of outcomes
☒	☐	Estimates of effect sizes (e.g. Cohen's *d*, Pearson's *r*), indicating how they were calculated

Our web collection on statistics for biologists contains articles on many of the points above.

Software and code

Policy information about availability of computer code

Data collection	We listed all software used in the experiments and for analysis in the Methods section. We used EthoVision XT software to detect and analyze the center point of each mouse to guide the delivery of light stimulation. We used a custom program in Microsoft Visual C++ for the MIDAS.
Data analysis	We mentioned all software used in the experiments and for analysis in the Methods section. We used the code related to the MIDAS system (https://github.com/Daegun-Kim/MIDAS) to control the mice's moving direction.

For manuscripts utilizing custom algorithms or software that are central to the research but not yet described in published literature, software must be made available to editors and reviewers. We strongly encourage code deposition in a community repository (e.g. GitHub). See the Nature Portfolio guidelines for submitting code & software for further information.

Data

Policy information about availability of data

All manuscripts must include a data availability statement. This statement should provide the following information, where applicable:

- Accession codes, unique identifiers, or web links for publicly available datasets
- A description of any restrictions on data availability
- For clinical datasets or third party data, please ensure that the statement adheres to our policy

All data that support the findings of this study are available from the corresponding authors upon request.

Human research participants

Policy information about studies involving human research participants and Sex and Gender in Research.

Reporting on sex and gender	This study did not involve human research participants.
Population characteristics	This study did not involve human research participants.
Recruitment	This study did not involve human research participants.
Ethics oversight	This study did not involve human research participants.

Note that full information on the approval of the study protocol must also be provided in the manuscript.

Field-specific reporting

Please select the one below that is the best fit for your research. If you are not sure, read the appropriate sections before making your selection.

☒ Life sciences ☐ Behavioural & social sciences ☐ Ecological, evolutionary & environmental sciences

For a reference copy of the document with all sections, see nature.com/documents/nr-reporting-summary-flat.pdf

Life sciences study design

All studies must disclose on these points even when the disclosure is negative.

Sample size	No statistical analysis was used to predetermine the sample size. Sample sizes were selected based on previous experience from related research and literature.
Data exclusions	No data were excluded.
Replication	Experimental findings were reliably reproduced among all subjects in all experiments comprised of multiple cohorts. Optogenetic experiments were conducted with more than 3 cohorts of animals.
Randomization	Animals were randomly assigned to control and treatment groups. The light-paired zone was randomized for a counterbalance for the animal with a place preference test.
Blinding	Investigators were not blinded to group allocation or data collection, but the analyses of behavioral data were performed blind with computer software.

Reporting for specific materials, systems and methods

We require information from authors about some types of materials, experimental systems and methods used in many studies. Here, indicate whether each material, system or method listed is relevant to your study. If you are not sure if a list item applies to your research, read the appropriate section before selecting a response.

Materials & experimental systems

n/a	Involved in the study	
☒	☐	Antibodies
☒	☐	Eukaryotic cell lines
☒	☐	Palaeontology and archaeology
☐	☒	Animals and other organisms
☒	☐	Clinical data
☒	☐	Dual use research of concern

Methods

n/a	Involved in the study	
☒	☐	ChIP-seq
☒	☐	Flow cytometry
☒	☐	MRI-based neuroimaging

Animals and other research organisms

Policy information about studies involving animals; ARRIVE guidelines recommended for reporting animal research, and Sex and Gender in Research

Laboratory animals	The male C57BL/6J mice had free access to food and water and were kept on a 12-h light-dark cycle. Experiments were performed on adult mice (9-11 weeks old).
Wild animals	The study did not involve wild animals.
Reporting on sex	Only male mice were used.
Field-collected samples	The study did not involve data collected from the field.
Ethics oversight	All care and handling of mice were performed according to the directives of the Animal Care and Use Committee of KAIST.

Note that full information on the approval of the study protocol must also be provided in the manuscript.
